# Supplementary material for: Benchtop micro-mashing: high-throughput, robust, experimental beer brewing
Source: Sci Rep. 2021 Jan 15;11:1480. doi: 10.1038/s41598-020-80442-7 (PMC7810850; doi:10.1038/s41598-020-80442-7)
Supplement: Supplementary file 2 — Supplementary Information 2. [file 41598_2020_80442_MOESM2_ESM.pdf]

Supplementary Material

Benchtop micro-mashing: high-throughput, robust, experimental beer brewing

Edward D. Kerr[1], Christopher H. Caboche[1], Peter Josh[1], and Benjamin L. Schulz[1*].

[1] School of Chemistry and Molecular Biosciences, The University of Queensland, St Lucia 4072, Queensland, Australia.

[*] To whom correspondence should be addressed: Benjamin L. Schulz, b.schulz@uq.edu.au, +61 7 3365 4875.

Supplementary Tables

Supplementary Table S1. Protein comparison: corrected p-values
Supplementary Table S2. Peptide comparison: corrected p-values
Supplementary Table S3. IAA peptide MSstats
Supplementary Table S4. NLTP1 peptide MSstats
Supplementary Table S5. MRM Targets
